# *Flavobacterium nakdongensis* sp. nov., Isolated from Fresh Water during the Cyanobacterial Bloom Period

**DOI:** 10.4014/jmb.2405.05026

**Published:** 2024-09-29

**Authors:** Ve Van Le, So-Ra Ko, Sang-Ah Lee, Chi-Yong Ahn

**Affiliations:** 1 Cell Factory Research Center, Korea Research Institute of Bioscience and Biotechnology (KRIBB), Daejeon 34141, Republic of Korea; 2 Faculty of Biotechnology, College of Applied Life Sciences, Jeju National University, Jeju 63243, Republic of Korea; 3 Department of Environmental Biotechnology, KRIBB School of Biotechnology, University of Science and Technology, Daejeon 34113, Republic of Korea

**Keywords:** Cyanobacterial bloom, novel species, *Flavobacterium nakdongensis*

## Abstract

A novel Gram-negative bacterial strain, 20NA77.7^T^, was isolated from fresh water of the Nakdong River. Strain 20NA77.7^T^ shared the highest similarity with *Flavobacterium indicum* GPTSA100-9^T^ (97.91%) and *Flavobacterium urocaniciphilum* DSM 27078^T^ (96.24%) in the 16S rRNA gene sequence. The digital DNA–DNA hybridization and average nucleotide identity values for strain 20NA77.7^T^ with *Flavobacterium* species were below 20.8% and 77.33%, respectively. The major fatty acids of strain 20NA77.7^T^ were identified as iso-C_15:0_, iso-C_16:0_, iso-C_15:1_ G, anteiso-C_15:0_, iso-C_15:0_ 3OH, and iso-C_16:0_ 3OH. Strain 20NA77.7^T^ contained phosphatidylethanolamine, one unidentified aminolipid, and three unidentified lipids as polar lipids and menaquinone-6 as menaquinone. The polyphasic evidence supports the classification of strain 20NA77.7^T^ as a novel species belonging to the genus *Flavobacterium*, for which the name *Flavobacterium nakdongensis* is proposed. The type strain is 20NA77.7^T^ (= KCTC 102000^T^ = LMG 33137^T^).

## Introduction

The genus *Flavobacterium* was first proposed by Bergey *et al*. [1] to accommodate the species of *Flavobacterium aquatile*. By April 2024, the genus *Flavobacterium* consists of 298 species with confirmed names [2]. Members of this genus inhabit various environments such as stream water [3], paddy fields [4], seawater [5], sediments [6], and soils [7]. *Flavobacterium* genus members have a broad spectrum of ecological roles and functional capabilities. They have been implicated as fish pathogens [8]. In addition to their role as pathogens, *Flavobacterium* species have been recognized for their ability to degrade various organic compounds, including diesel fuel, which makes them potential candidate for bioremediation applications [9]. Certain species within the genus are recognized for their ability to synthesize auxins, an important plant growth hormone [4], and carotenoids, which are pigments that provide protection against oxidative stress [10].

Cyanobacteria are key components of aquatic ecosystems, providing organic carbon to other organisms through photosynthesis [11]. Nevertheless, the rapid proliferation of cyanobacteria, known as cyanobacterial blooms, threatens ecosystems and public health [12]. Cyanobacteria exhibit diverse interactions with heterotrophic bacteria, ranging from mutualistic to competitive relationships. Some bacteria support cyanobacterial growth by synthesizing vitamins and cofactors [13]. However, certain bacteria can inhibit the growth of cyanobacteria by directly attacking and producing algicidal compounds [14]. Therefore, heterotrophic bacteria are considered a key player in the formation and decline of cyanobacterial blooms [15, 16].

The *Flavobacterium* genus is widely distributed in the phycosphere of cyanobacteria. They are abundant in this ecological niche and have been identified as key indicators of cyanobacterial blooms [16]. *Flavobacterium* species are known to produce carotenoids, which provide photoprotection to cyanobacteria against high-intensity sunlight, contributing to the widespread cyanobacterial blooms [17]. This is especially significant in habitats where cyanobacteria are exposed to strong sunlight, such as in shallow waters or during algal blooms. In the course of investigating the relationship between cyanobacteria and bacterial communities, strain 20NA77.7^T^ was isolated from Nakdong River during a cyanobacterial bloom. This study investigated the taxonomic position of the newly isolated bacterial strain.

## Materials and Methods

### Conditions for Isolation of Strain 20NA77.7^T^

A freshwater sample was obtained from the surface water of Nakdong River at the Haman site (35o 22' 39.42'' N, 128o 33' 15.96'' E) in July 7, 2020. To isolate the strain, it was serially diluted in sterilized distilled water and then inoculated onto R2A agar. The plates were subsequently incubated at 25°C for 7 days. Following this incubation, a distinct yellow colony was selected and transferred to fresh R2A agar medium to ensure purity. A pure culture was achieved through a process of restreaking. Thus purely isolated strain was preserved at -80°C in R2A liquid culture with 20% glycerol as a cryoprotectant to maintain viability for subsequent studies. In the course of our study, we chose *Flavobacterium indicum* DSM 17447^T^ [18] and *Flavobacterium urocaniciphilum* DSM 27078^T^ [19] as reference strains for comparative polyphasic characterization. This selection was based on the sequence similarity observed in the 16S rRNA gene, as well as the finding from our phylogenetic analysis. Since strain 20NA77.7^T^ and the reference strains exhibited growth in R2A medium, they were cultured on this medium for all experiments unless otherwise stated.

### Phylogenetic Analysis and Genomic Characteristics

Genomic DNA was extracted using the FastDNA Spin DNA extraction kit, followed by the amplification of the 16S rRNA gene and sequencing. All procedures were performed according to the protocols established by Le *et al*.[20]. To analyze the phylogenetic relationships, the 16S rRNA gene sequence from all species that have been validly published in the EzBioCloud (www.ezbiocloud.net) were used [21]. Based on these alignments, phylogenetic trees were built using the MEGA 11 software [22] with neighbor-joining [23], minimum evolution [24], and maximum likelihood [25] methods. The Kimura’s two-parameter model was applied to determine the genetic distances [26]. A bootstrap trial consisting of 1,000 replicates was conducted to assess the robustness and reliability of the phylogenetic trees. Reference strains were chosen according to the phylogenetic trees created from the 16S rRNA gene sequence data, which showed that *Flavobacterium indicum* DSM 17447^T^ and *Flavobacterium urocaniciphilum* DSM 27078^T^ were highly similar to the target strain.

The genome sequence of strain 20NA77.7^T^ was obtained using the PacBio Sequel II system and Illumina HiSeq sequencing system (Macrogen Inc., Republic of Korea) and assembled as described previously [27]. To verify the integrity of the assembled genome, potential contamination was evaluated using the ContEst16S tool [28]. For genome annotation, we utilized several tools including Prokka v.1.12, Rapid Annotation Subsystem Technology (RAST), and NCBI Prokaryotic Genome Annotation Pipeline (PGAP) [29–31]. In addition, we calculated the average nucleotide identity (ANI) values to determine the genomic relatedness and taxonomic affiliation of the strains. The digital DNA-DNA hybridization (dDDH) was also determined to further confirm its taxonomic position. These calculations were carried out using ANI Calculator [32] and the Genome-to-Genome Distance Calculator supplemented with the recommended formula 2 [33], respectively. Type (Strain) Genome Server was utilized to generate a phylogenomic tree for strain 20NA77.7^T^ [34]. AntiSMASH version 7.0 was used to predict biosynthetic gene clusters, with the detection strictness parameter set as ‘strict’ [35]. The dbCAN3 meta server was used to find the genes encoding carbohydrate-active enzymes (CAZymes) [36]. Only genes identified by all three tools (DIAMOND, HMMER, and dbCAN-sub) were taken into consideration.

### Physiological and Chemotaxonomical Characterization

The morphology of strain 20NA77.7^T^ cells was noted using transmission electron microscopy (CM-20, Philips, The Netherlands), and Gram staining was conducted with a Gram staining kit from bioMérieux. The tolerable ranges for the growth of strain 20NA77.7^T^ were determined for pH and temperature, according to Le *et al*. [37]. The impact of different media on the growth of strain 20NA77.7^T^ was evaluated using R2A agar, Luria-Bertani (LB, Difco, USA), nutrient agar (NA, Difco), and tryptone soy agar (TSA, Difco) at 25°C over a 3-day incubation period. The salt tolerance range was tested for strain 20NA77.7^T^ on R2A agar medium added with nine different concentrations of NaCl: 0.1, 0.3, 0.5, 1.0, 1.5, 2.0, 2.5, 3.0, and 3.5%. Catalase activity was verified by observing oxygen formation after adding a 3% (v/v) solution of H_2_O_2_. Oxidase activity was assessed using a 1% aqueous solution of *N*, *N*, *N’*, *N’*-tetramethyl-1,4-phenylenediamine (Sigma, USA). API 50CH, API ZYM, and API 20NE kits (bioMérieux, France) were used to determine physiological and biochemical properties. Hydrolysis of skim milk and lipids were tested following the method of Smibert and Krieg [38]. The flexirubin-type pigment was measured by adding a 20% (w/v) solution of KOH to bacterial cells [39].

The cells of strain 20NA77.7^T^ and closely related type strains were harvested on R2A medium at temperature of 25°C over a period of 3 days. The composition of cellular fatty acids was decided using the MIDI (Microbial Identification System, Sherlock, USA), and the cellular polar lipids were separated and detected from the freeze-dried cells as previously described [3]. Aminolipids was identified using ninhydrin. Quinones were obtained through the extraction process using a chloroform:methanol mixture solution (2:1, v/v) by stirring wet culture pellets and analyzed using high-performance liquid chromatography (YL9100, Young Lin, Republic of Korea)[40].

## Results and Discussion

### Phylogeny based on 16S rRNA Gene Sequences

**Fig. 1:**
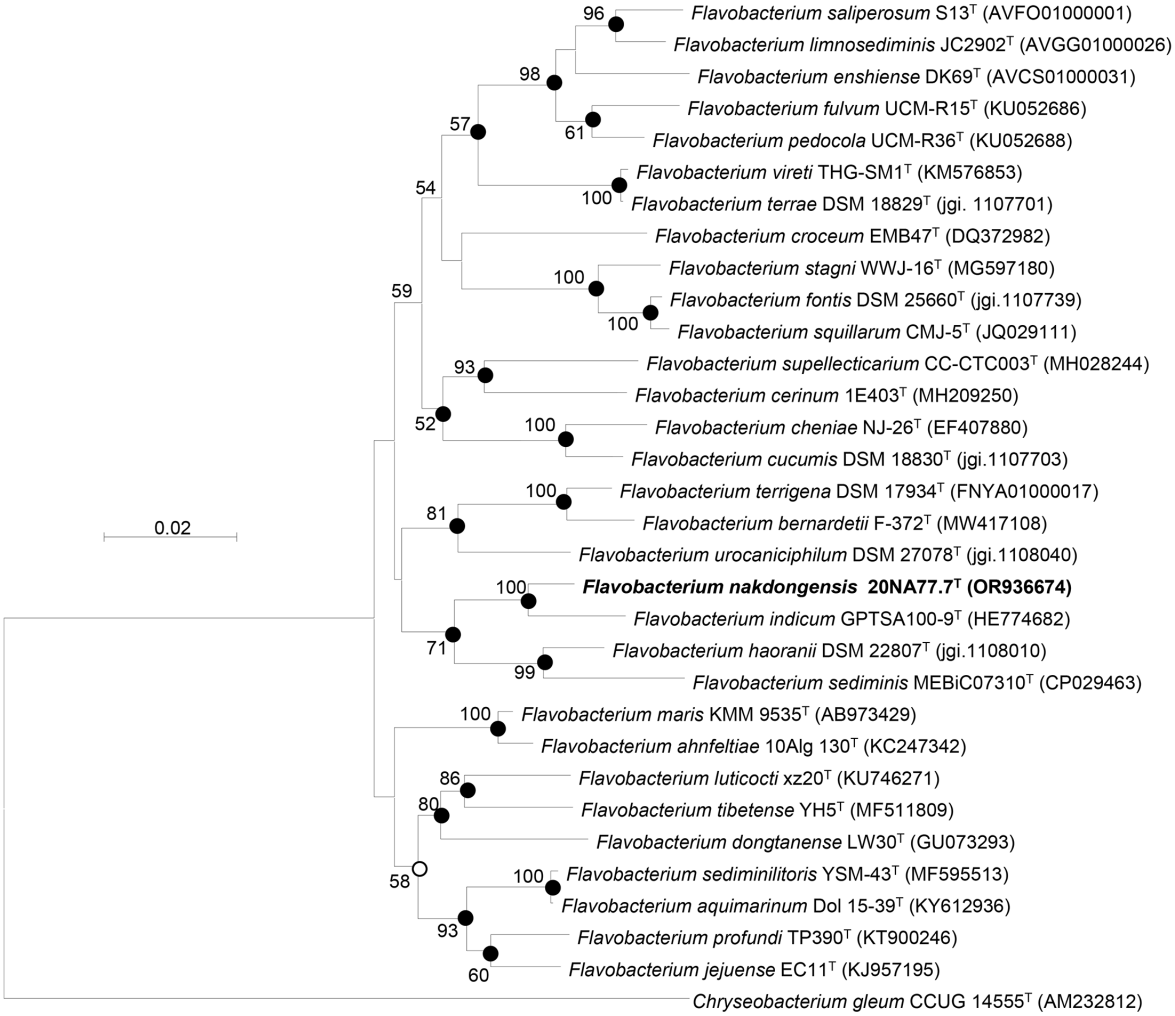
Phylogenetic tree for strain 20NA77.7^T^ built by using the neighbor-joining method for 16S rRNA gene sequences. At each branch point, bootstrap value (≥ 50%) based on 1,000 replications is noted. Filled circles at nodes indicate that the corresponding nodes were also observed in the trees built with other algorithms (NJ, ME, and ML methods). However, open circles at nodes imply that they appeared only with NJ and ML algorithms. *Chryseobacterium gleum* CCUG 14555^T^ (AM232812) was used as an outgroup. Scale bar, 0.02 nucleotide substitutions per nucleotide position.

Strain 20NA77.7^T^ shared the top similarity with *Flavobacterium indicum* GPTSA100-9^T^ (97.91%) and *Flavobacterium urocaniciphilum* DSM 27078^T^ (96.24%), respectively. Other *Flavobacterium* species exhibited less than 96.0%. These values were less than 98.7% for demarcating bacterial species, suggesting that the strain can be classified into separate species [41]. In the phylogenetic trees, strain 20NA77.7^T^ developed a robust cluster with *Flavobacterium indicum* GPTSA100-9^T^, further verified by 100% of bootstrap values (Figs. 1, S1, and S2).

### Genomic and Phylogenomic Analyses

**Fig. 2:**
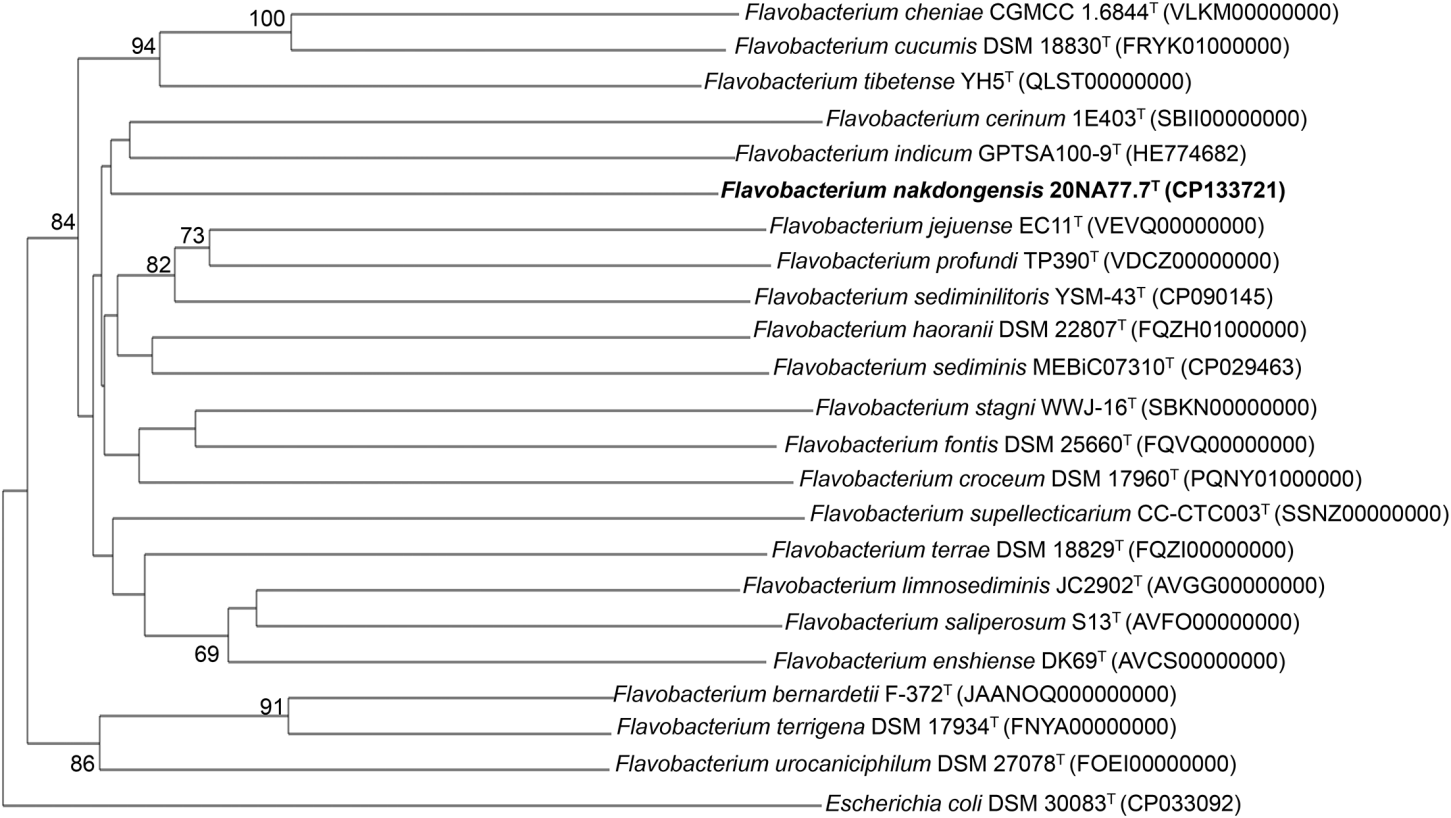
Phylogenomic tree for strain 20NA77.7^T^ and closely related taxa. The branch lengths are proportion to the GBDP distance formula d5. The numbers denoted above the branch points are GBDP pseudo-bootstrap support values (> 60% from 100 replications). An average branch support was 48.6%. The tree was rooted at the midpoint.

A single contig consisting of 2,331,026 bases was assembled for strain 20NA77.7^T^, with a GC content of 32.54%. The PGAP annotation of the genome revealed 1,995 coding sequences, 9 rRNAs, 44 tRNAs, and 4 ncRNAs (Table S1). The completeness of genome assembly and sequencing coverage depth were 100% and 805.35×, respectively. The two sequences of 16S rRNA gene obtained from Sanger sequencing (1,352 bp) and whole-genome sequencing (1,516 bp) exhibited complete similarity. Three 16S rRNA gene segments within the whole-genome sequence showed 100% similarity, confirming the accuracy of the genome data [28]. The ANI and dDDH values of strain 20NA77.7^T^ with *Flavobacterium* species were below 77.33 and 20.8%, respectively (Table S2). Such values are considerably lower than the recommended thresholds for species demarcation, with ANI below 95–96% [32] and dDDH below 70.0% [33], suggesting that the strain is a novel species of the genus *Flavobacterium*. The phylogenomic tree further confirmed the taxonomic position of strain 20NA77.7^T^ within the genus *Flavobacterium* (Fig. 2). The high relative abundance of genes in the genome of 20NA77.7^T^ was involved in crucial cellular functions such as “protein metabolism”, “RNA metabolism”, and “cofactors, vitamins, prosthetic groups, pigments” (Fig. S3).

Bloom-forming cyanobacteria not only influence the composition of microbial communities but also rely on the beneficial functions of their associated microbes [13]. Although *Flavobacterium* species have been identified as key indicators of cyanobacterial blooms and are known to be abundant in the phycosphere of cyanobacteria [17], their exact contribution to these blooms remains unclear. The complex organic matter can be degraded by CAZymes [42]. The genome of strain 20NA77.7^T^ contains a wide variety of CAZymes, suggesting that it can utilize organic matter released from cyanobacterial cells (Table S3) [43]. For example, GH23 can break down peptidoglycan, while GH31 and GH53 can degrade cellulose and hemicellulose [44]. Strain 20NA77.7^T^ harbors three gene clusters that biosynthesize two terpenes and one arylpolyene, with one of the terpenes predicted to be carotenoid (Table S4). The carotenoids produced by *Flavobacterium* could provide photoprotection for cyanobacteria, potentially enhancing its ability to tolerate highly intense sunlight [17]. Cyanobacteria acquire vitamin B via mutualistic interactions with heterotrophic bacteria [45]. Since iron influences respiration and photosynthesis of cyanobacteria, shortage of iron could inhibit the development of cyanobacterial blooms [46, 47]. Strain 20NA77.7^T^ possessed the complete pathway of heme and biotin (Vitamin B7) biosynthesis, suggesting its potential role in promoting cyanobacterial blooms (Table S5).

### Morphological and Phenotypic Analyses

Cells of strain 20NA77.7^T^ were observed to be rod-shaped (0.4–1.1 μm long and 0.2–0.3 μm wide), Gram-stain-negative, and non-spore-forming (Fig. S4). The colonies grown on the R2A agar medium were circular, convex, smooth, and yellow-colored. The strain was positive for oxidase and catalase activities. The growth of 20NA77.7^T^ was observed at 15-30°C (optimum 25°C), pH 6.5-7.5 (optimum pH 7.0), and 0-0.5% NaCl (optimum 0% NaCl). The strain can grow on R2A agar medium but not on TSA, NA, and LB media. It was differentiated from the selected reference strains by the production possibility of esterase (C4). Upon the addition of a 20% KOH (w/v) solution, the color change of the colonies was not observed, suggesting that this strain does not produce flexirubin-type pigments. Details on the differential phenotypic characteristics of 20NA77.7^T^ and its intimate type strains are given in Table 1.

### Chemotaxonomic Characteristics

The major cellular fatty acids of strain 20NA77.7^T^ (> 5% of total fatty acids) were iso-C_15:0_ , iso-C_16:0_, iso-C_15:1_ G, anteiso-C_15:0_ , iso-C_15:0_ 3OH, and iso-C_16:0_ 3OH (Table 2). The presence of C_17:0_ 2OH distinguished 20NA77.7^T^ from its close type strains. The main respiratory quinones of strain 20NA77.7^T^ was menaquinone-6 (MK-6), which is consistent with the characteristics described for the genus *Flavobacterium*. The polar lipids present in strain 20NA77.7^T^ were phosphatidylethanolamine, one unidentified aminolipid, and three unidentified lipids (Fig. S5). Phosphatidylethanolamine was a major polar lipid and this result aligned with earlier descriptions of *Flavobacterium* species [3, 4, 48].

### Taxonomic Conclusions

Phylogenetic, chemotaxonomic, and phenotypic analyses suggested that strain 20NA77.7^T^ belongs to the genus *Flavobacterium*. The low genomic relatedness, along with distinctive phenotypic properties and fatty acid profiles observed between this strain and other *Flavobacterium* species, support the proposal that strain 20NA77.7^T^ is a novel species within the genus *Flavobacterium*, for which the name *Flavobacterium nakdongensis* is proposed.

### Description of *Flavobacterium nakdongensis* sp. nov.

*Flavobacterium nakdongensis* (nak.dong.en'sis. N.L. masc. adj. *nakdongensis* pertaining to Nakdong River, from where the type strain was isolated)

Cells are Gram-negative and rod-shaped (0.4–1.1 μm long and 0.2–0.3 μm wide). Colonies grown on R2A medium for two days are convex, smooth, and yellow-colored. Growth occurs in the range of 15–30°C and pH 6.5–7.5. Does not require NaCl for growth but can tolerate up to 0.5%. Flexirubin-type pigments are not synthesized. Positive for diverse enzyme activities of trypsin, esterase lipase (C8), alkaline phosphatase, valine arylamidase, esterase (C4), leucine arylamidase, naphthol-AS-BI-phosphohydrolase, cystine arylamidase, and acid phosphatase (API ZYM test strip). Also positive for hydrolysis of esculin (API 20NE test). MK-6 is the key respiratory quinone. Phosphatidylethanolamine, one unidentified aminolipid, and three unidentified lipid are the main polar lipids. The G+C content of the type strain is 32.54%, determined by genomic sequence.

The type strain, 20NA77.7^T^ (= KCTC 102000^T^ = LMG 33137^T^), was isolated from the surface water of Nakdong River when severe cyanobacterial blooms occurred. The NCBI accession numbers are OR936674 for 16S rRNA gene and CP133721 for genome sequence.

## Supplemental Materials

Supplementary data for this paper are available on-line only at http://jmb.or.kr.



## Figures and Tables

**Table 1:** Differential characteristics of strain 20NA77.7^T^ and the closely related species of the genus *Flavobacterium*.

Characteristics	1	2	3	4^†^
Motility	-	-	-	+
Growth range				
pH	6.5-7.5	5.0-11.0*	6.5-8.5**	6.0-9.0
Temperature (°C)	15-30	15-42*	10.0-37.0**	4-37
NaCl (%, w/v)	0-0.5	0-2.0*	0-0.5**	0-2.0
API ZYM test				
Esterase (C4)	+	-	-	-
Esterase lipase (C8)	+	+	-	-
Cystine arylamidase	+	+	-	-
α-chymotrypsin	-	-	+	-
Acid phosphatase	+	-	+	+
API 20NE test				
Hydrolysis of gelatin	-	+	-	+
Hydrolysis of				
Tween 80	-	+	-	-
Genome size (Mb)	2.3	3.0	2.8	4.1
Polar lipids	PE, AL, 3L	nd	PE, APL, 5AL, 4L**	PE, 4AL, 2 PL

Strains: 1, 20NA77.7^T^; 2, *Flavobacterium indicum* DSM 17447^T^; 3, *Flavobacterium urocaniciphilum* DSM 27078^T^; 4, *Flavobacterium cerinum* 1E403^T^. +, positive; -, negative. All data were obtained during this study, except where indicated otherwise. *Data were obtained from Saha *et al*. [18]; **Data were obtained from Fujii *et al*. [19]; ^†^Data were obtained from Zhang *et al*. [48]. nd, no data; PE, phosphatidylethanolamine; AL, unidentified aminolipid; APL, aminophospholipid; PL, phospholipid; L, unidentified lipid.

**Table 2:** Cellular fatty acid contents (%) of strain 20NA77.7^T^ and related species of the genus *Flavobacterium*.

Fatty acid (%)	1	2	3	4^†^
iso-C_13:0_	2.4	**5.0**	1.4	nd
iso-C_14:0_	4.6	3.0	-	tr
iso-C_14:0_ 3OH	1.2	2.2	1.9	nd
C_15:0_ 2OH	tr	2.9	tr	1.7
C_15:0_ 3OH	2.9	-	3.0	nd
C_15:1_ *ω*6*c*	2.4	2.6	4.9	1.6
iso-C_15:0_	**19.6**	**15.2**	**19.1**	**13.4**
iso-C_15:0_ 3OH	**5.5**	**12.5**	**11.4**	**4.2**
iso-C_15:1_ G	**17.4**	**15.9**	**13.9**	**5.3**
anteiso-C_15:1_ A	2.0	tr	-	nd
anteiso-C_15:0_	**8.1**	-	-	4.4
C_16:0_	1.0	tr	1.0	**5.7**
C_16:0_ 3OH	1.1	2.0	2.5	3.4
iso-C_16:0_	**9.8**	2.5	4.8	5.2
iso-C_16:0_ 3OH	**9.1**	**5.2**	4.0	4.3
iso-C_16:1_ H	-	-	2.9	2.2
C_17:1_ *ω*6*c*	tr	-	2.4	nd
C_17:0_ 2OH	1.0	-	-	nd
iso-C_17:0_ 3OH	4.9	**11.0**	**11.9**	**8.5**
**Summed feature** *				
3	tr	**12.50**	**7.10**	**24.3**
9	tr	2.10	3.50	nd

Strains: 1, 20NA77.7^T^; 2, *Flavobacterium indicum* DSM 17447^T^; and 3, *Flavobacterium urocaniciphilum* DSM 27078^T^; 4, *Flavobacterium cerinum* 1E403^T^. All data were obtained in this study, except where indicated otherwise. ^†^Data were obtained from Zhang *et al*. [48]. Only fatty acids that accounted for > 1% in at least one of the strains are listed. Major components (> 5.0%) are highlighted in bold; tr, trace amount (< 1.0%); –, not detected; nd, no data. *Summed features are fatty acids that cannot be resolved reliably from another fatty acid using the chromatographic conditions chosen. The MIDI system groups these fatty acids together as one feature with a single percentage of the total. *Summed feature 3 consisted of C_16:1_*ω*7*c* and/or C_16:1_*ω*6*c*; Summed feature 9 consisted of iso-C_17:1_ *ω*9*c* and/or 10-methyl C_16:0_.
