# Supplementary material for: Flavobacterium nakdongensis sp. nov., Isolated from Fresh Water during the Cyanobacterial Bloom Period
Source: J Microbiol Biotechnol. 2024 Sep 29;34(11):2245–51. doi: 10.4014/jmb.2405.05026 (PMC11637836; doi:10.4014/jmb.2405.05026)
Supplement: Supplementary file 1 [file jmb-34-11-2245-supple.pdf]

J. Microbiol. Biotechnol. 2024. 34(11): 2245–2251

https://doi.org/10.4014/jmb.2405.05026

Supplementary Tables and Figures

Flavobacterium nakdongensis sp. nov., isolated from fresh Water during the cyanobacterial bloom period

Ve Van Le[1], So-Ra Ko[1], Sang-Ah Lee[2], and Chi-Yong Ahn[1,3*]

[1]Cell Factory Research Center, Korea Research Institute of Bioscience and Biotechnology (KRIBB), 125 Gwahak-ro, Yuseong-gu, Daejeon 34141, Republic of Korea

[2]Faculty of Biotechnology, College of Applied Life Sciences, Jeju national University, Jeju 63243, Republic of Korea

[3]Department of Environmental Biotechnology, KRIBB School of Biotechnology, University of Science and Technology, Daejeon 34113, Republic of Korea

***Correspondence: Chi-Yong Ahn**

E-mail: cyahn@kribb.re.kr

Table S1. Genome features of strain 20NA77.7^T

Features	20NA77.7^T
Genome size	2.3 Mb
Number of chromosomes	1
Contig N50	2.3 Mb
Contig L50	1
Genes (total)	2052
CDSs (total)	1995
Genes (coding)	1988
RNA	57
rRNAs	9
tRNAs	44
ncRNAs	4
Pseudo Genes	7
CDS (without protein)	7

Table S2. Genome comparison of strain 20NA77.7^{T} with reference genomes of related taxa.

Species	Strain	Accession No.	ANI (%)	dDDH (%)
Flavobacterium saliperosum	S13^{T}	AVFO00000000	72.26	20.4
Flavobacterium limnosediminis	JC2902^{T}	AVGG00000000	72.27	20.7
Flavobacterium enshiense	DK69^{T}	AVCS00000000	72.09	20.4
Flavobacterium terrae	DSM 18829^{T}	FQZI00000000	73.32	20.0
Flavobacterium croceum	DSM 17960^{T}	PQNY01000000	72.69	19.2
Flavobacterium stagni	WWJ-16^{T}	SBKN00000000	71.52	19.6
Flavobacterium fontis	DSM 25660^{T}	FQVQ00000000	71.07	21.1
Flavobacterium supellecticarium	CC-CTC003^{T}	SSNZ00000000	71.50	20.7
Flavobacterium cerinum	1E403^{T}	SBII00000000	70.30	20.6
Flavobacterium cheniae	CGMCC 1.6844^{T}	VLKM00000000	74.89	20.7
Flavobacterium cucumis	DSM 18830^{T}	FRYK01000000	74.59	20.4
Flavobacterium terrigena	DSM 17934^{T}	FNYA00000000	75.73	20.2
Flavobacterium bernardetii	F-372^{T}	JAANOQ000000000	75.84	20.4
Flavobacterium urocaniciphilum	DSM 27078^{T}	FOEI00000000	75.80	20.3
Flavobacterium indicum	GPTSA100-9^{T}	HE774682	77.33	20.8
Flavobacterium haoranii	DSM 22807^{T}	FQZH01000000	74.46	19.6
Flavobacterium sediminis	MEBiC07310^{T}	CP029463	72.94	20.3
Flavobacterium tibetense	YH5^{T}	QLST00000000	74.43	20.6
Flavobacterium sediminilitoris	YSM-43^{T}	CP090145	74.46	20.3
Flavobacterium profundi	TP390^{T}	VDCZ00000000	74.04	20.3
Flavobacterium jejuense	EC11^{T}	VEVQ00000000	74.46	19.5

Table S3. List of carbohydrate-active enzymes (CAZymes) in the genome of strain 20NA77.7^{T}. GH, glycoside hydrolase; GT, glycosyltransferase; CE, carbohydrate esterase; CBM, carbohydrate-binding module.

Gene ID	EC#	HMMER	dbCAN_sub	DIAMOND
20NA_77_7_1_00014	-	GT2(6-166)	GT2	GT2
20NA_77_7_1_00022	-	GT4(193-340)	GT4_e3703	GT4
20NA_77_7_1_00033	-	GT9(61-290)	GT9_e87	GT9
20NA_77_7_1_00046	-	GT51(64-243)	GT51_e181	GT51
20NA_77_7_1_00134	-	GT2(6-170)	GT2	GT2
20NA_77_7_1_00233	-	GT5(5-235)	GT5_e28	GT5
20NA_77_7_1_00327	-	GT30(16-178)	GT30_e55	GT30
20NA_77_7_1_00383	-	GT28(192-349)	GT28_e0	GT28
20NA_77_7_1_00526	-	GT2(46-199)	GT2	GT2
20NA_77_7_1_00596	-	GT2(4-188)	GT2	GT2
20NA_77_7_1_00631	-	CBM4(20-144)	CBM4_e19	CBM4
20NA_77_7_1_00635	-	GH16_3(41-270)	GH16_e233	GH16_3
20NA_77_7_1_00636	3.2.1.21|3.2.1.-	GH3(107-328)	GH3_e1	GH3
20NA_77_7_1_00637	3.2.1.39	GH16_3(33-277)	GH16_e273	GH16_3
20NA_77_7_1_00654	-	GT27(7-233)	GT2	GT2
20NA_77_7_1_00664	-	CE14(17-121)	CE14_e27	CE14
20NA_77_7_1_00747	-	GT4(200-344)	GT4_e858	GT4
20NA_77_7_1_00749	-	GT2(5-106)	GT2	GT2
20NA_77_7_1_00751	-	GT2(6-142)	GT2	GT2
20NA_77_7_1_00767	-	GT4(225-386)	GT4_e535	GT4
20NA_77_7_1_00768	-	GH130_14(7-337)	GH130_e12	GH130

20NA_77_7_1_00841	-	GH53(35-351)	GH53_e13	GH53
20NA_77_7_1_00969	-	CE11(4-238)	CE11_e18	CE11
20NA_77_7_1_00986	-	GT4(196-342)	GT4_e2566	GT4
20NA_77_7_1_00988	-	GH3(94-320)	GH3_e104	GH3
20NA_77_7_1_01212	3.2.1.-|3.2.1.113|3.2.1.114|3.2.1.24	GH92(226-717)	GH92_e0	GH92
20NA_77_7_1_01258	-	GH23(86-224)	GH23_e357	GH23
20NA_77_7_1_01261	-	GT2(5-171)	GT2	GT2
20NA_77_7_1_01263	-	GT4(200-347)	GT4_e823	GT4
20NA_77_7_1_01289	-	GT2(4-136)	GT2	GT2
20NA_77_7_1_01307	-	GT2(4-133)	GT2	GT2
20NA_77_7_1_01392	3.2.1.-|3.2.1.113|3.2.1.114|3.2.1.24	GH92(231-667)	GH92_e0	GH92
20NA_77_7_1_01537	-	GT4(199-353)	GT4_e2685	GT4
20NA_77_7_1_01593	-	GH171(92-436)	GH171_e3	GH171
20NA_77_7_1_01670	3.2.1.49	GH109(39-454)	GH109_e1	GH109
20NA_77_7_1_01676	-	GT2(5-142)	GT2	GT2
20NA_77_7_1_01725	3.2.1.52|3.2.1.-	GH20(145-491)	GH20_e12	GH20
20NA_77_7_1_01729	3.2.1.25	GH2(20-740)	GH2_e80	GH2
20NA_77_7_1_01866	-	GT19(4-352)	GT19_e2	GT19
20NA_77_7_1_01905	3.2.1.1	GH13(55-348)	GH13_e187	GH13
20NA_77_7_1_01906	2.4.1.161	GH31(234-668)	GH31_e76	GH31
20NA_77_7_1_01908	3.2.1.1	GH13_46(154-477)	GH13_e235	GH13_46
20NA_77_7_1_01909	2.4.1.8	GH65(325-706)	GH65_e0	GH65
20NA_77_7_1_01916	-	GH13_10(407-574)	CBM48_e54+GH13_e156	GH13
20NA_77_7_1_02033	-	GH23(138-274)	GH23_e731+CBM50_e954	CBM50+GH23

Table S4. List of putative secondary metabolite-producing biosynthetic clusters as predicted by antiSMASH.

Region	Type	From	To	Most similar known cluster
Region 1	terpene	186091	207236	
Region 2	arylpolyene	261353	302498	
Region 3	terpene	844012	864851	carotenoid

Table S5. Complete metabolic pathways in the genome of strain 20NA77.7^T predicted by BlastKOALA.

Functional category	Pathway module	Module	Description
Carbohydrate metabolism	Central carbohydrate metabolism	M00002	Glycolysis, core module involving three-carbon compounds
		M00003	Gluconeogenesis, oxaloacetate => fructose-6P
		M00307	Pyruvate oxidation, pyruvate => acetyl-CoA
		M00010	Citrate cycle, first carbon oxidation, oxaloacetate => 2-oxoglutarate
		M00007	Pentose phosphate pathway, non-oxidative phase, fructose 6P => ribose 5P
		M00005	PRPP biosynthesis, ribose 5P => PRPP
Energy metabolism	ATP synthesis	M00144	NADH:quinone oxidoreductase, prokaryotes
		M00155	Cytochrome c oxidase, prokaryotes
		M00156	Cytochrome c oxidase, cbb3-type
		M00157	F-type ATPase, prokaryotes and chloroplasts
Lipid metabolism	Fatty acid metabolism	M00082	Fatty acid biosynthesis, initiation
		M00083	Fatty acid biosynthesis, elongation
		M00086	beta-Oxidation, acyl-CoA synthesis
	Lipid metabolism	M00093	Phosphatidylethanolamine (PE) biosynthesis, PA => PS => PE
Nucleotide metabolism	Purine metabolism	M00048	De novo purine biosynthesis, PRPP + glutamine => IMP
		M00049	Adenine ribonucleotide biosynthesis, IMP => ADP,ATP

		M00050	Guanine ribonucleotide biosynthesis, IMP => GDP,GTP
		M00053	Deoxyribonucleotide biosynthesis, ADP/GDP/CDP/UDP => dATP/dGTP/dCTP/dUTP
	Pyrimidine metabolism	M00052	Pyrimidine ribonucleotide biosynthesis, UMP => UDP/UTP,CDP/CTP
Amino acid metabolism	Serine and threonine metabolism	M00621	Glycine cleavage system
	Branched-chain amino acid metabolism	M00019	Valine/isoleucine biosynthesis, pyruvate => valine / 2-oxobutanoate => isoleucine
		M00570	Isoleucine biosynthesis, threonine => 2-oxobutanoate => isoleucine
	Arginine and proline metabolism	M00970	Proline degradation, proline => glutamate
	Histidine metabolism	M00045	Histidine degradation, histidine => N-formiminoglutamate => glutamate
Glycan metabolism	Lipopolysaccharide metabolism	M00063	CMP-KDO biosynthesis
Metabolism of cofactors and vitamins	Cofactor and vitamin metabolism	M00120	Coenzyme A biosynthesis, pantothenate => CoA
		M00123	Biotin biosynthesis, pimeloyl-ACP/CoA => biotin
		M00881	Lipoic acid biosynthesis, plants and bacteria, octanoyl-ACP => dihydrolipoyl-E2/H
		M00121	Heme biosynthesis, plants and bacteria, glutamate => heme

Biosynthesis of terpenoids and polyketides	Terpenoid backbone biosynthesis	M00364	C10-C20 isoprenoid biosynthesis, bacteria
	Polyketide sugar unit biosynthesis	M00793	dTDP-L-rhamnose biosynthesis

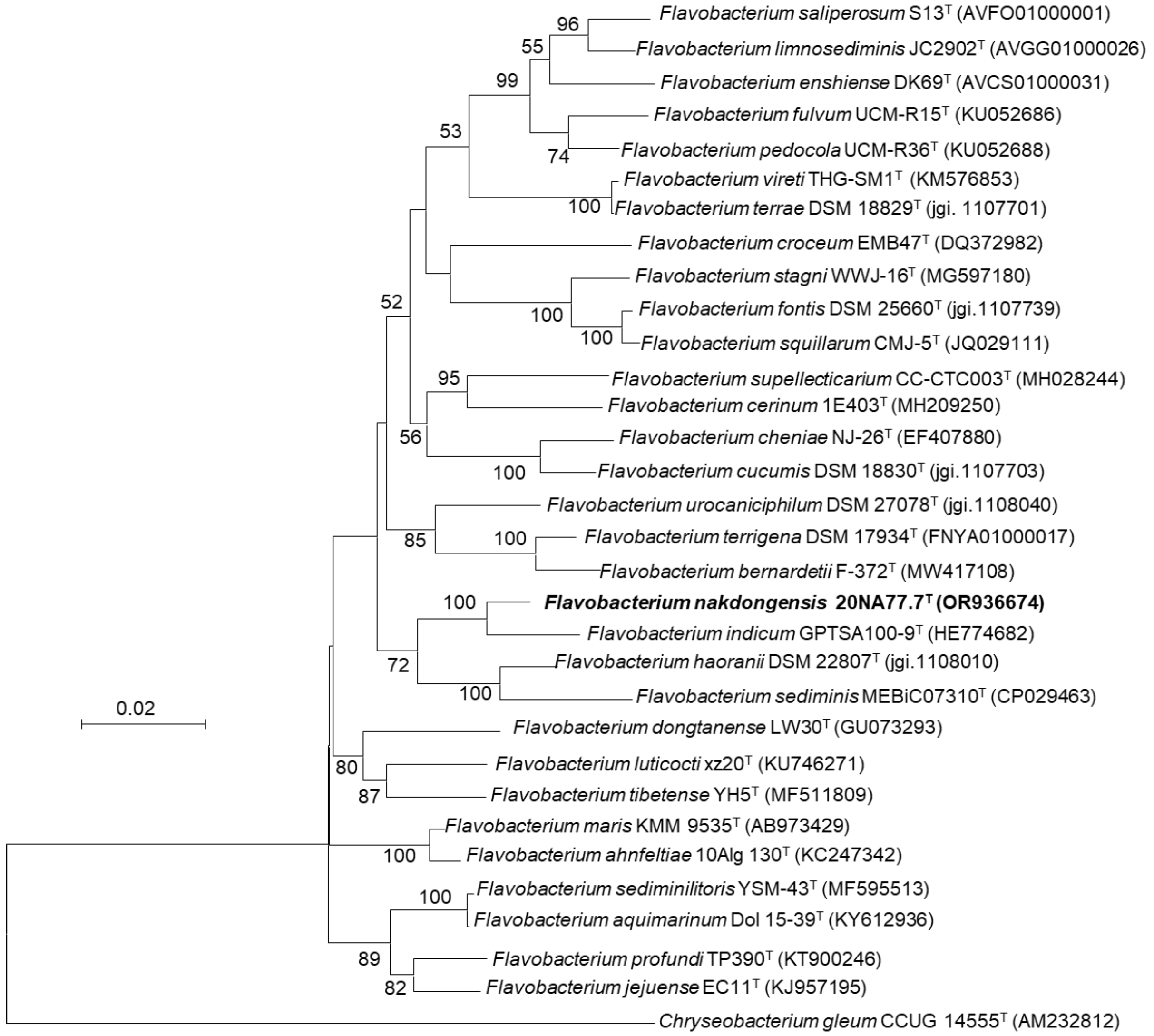

Fig. S1. **Minimum-evolution phylogenetic tree based on the 16S rRNA gene sequences depicting the position of strain 20NA77.7^T among the related *Flavobacterium* species.** Bootstrap values (≥ 50%) based on 1,000 replications are shown at branch points.

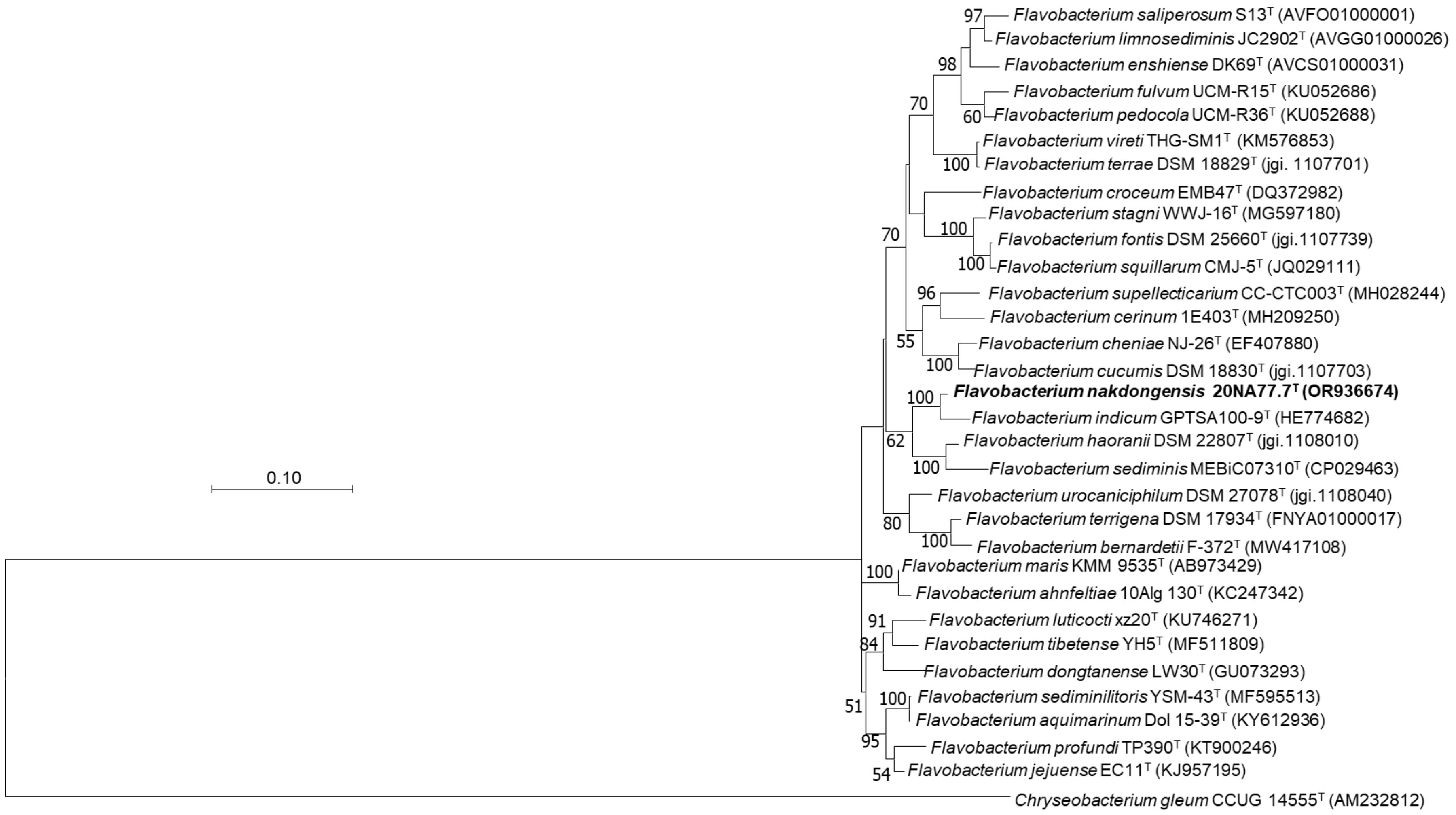

Fig. S2. **Maximum-likelihood phylogenetic tree based on the 16S rRNA gene sequences depicting the position of strain 20NA77.7^{T} among the related *Flavobacterium* species. Bootstrap values (≥ 50%) based on 1,000 replications are shown at branch points.**

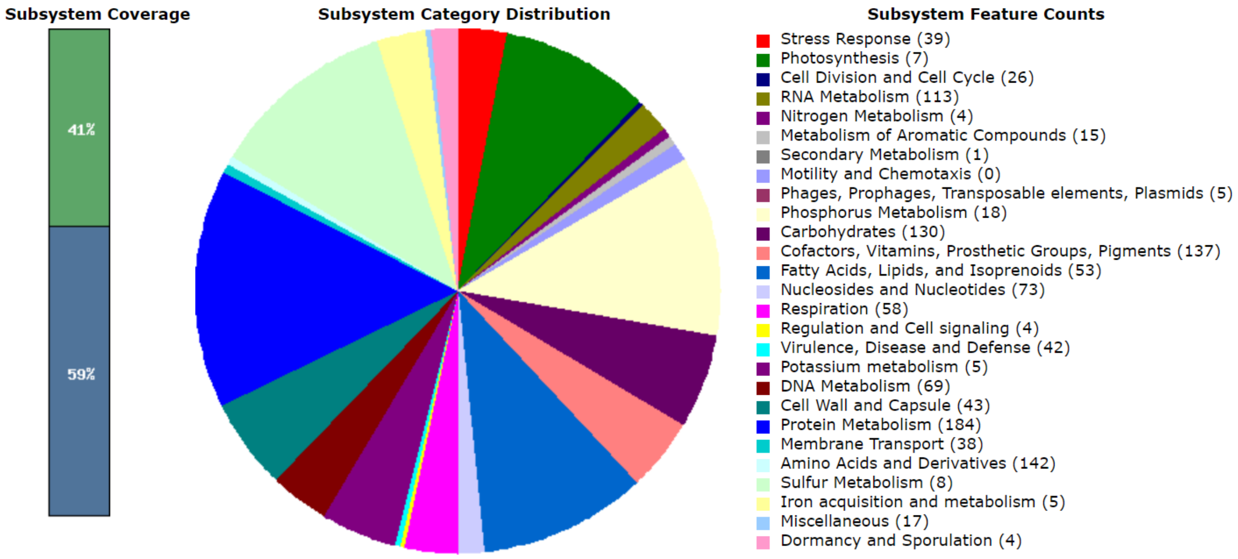

Fig. S3. Subsystem categories and features distribution of strain 20NA77.7^{T} genome using the RAST annotation server.

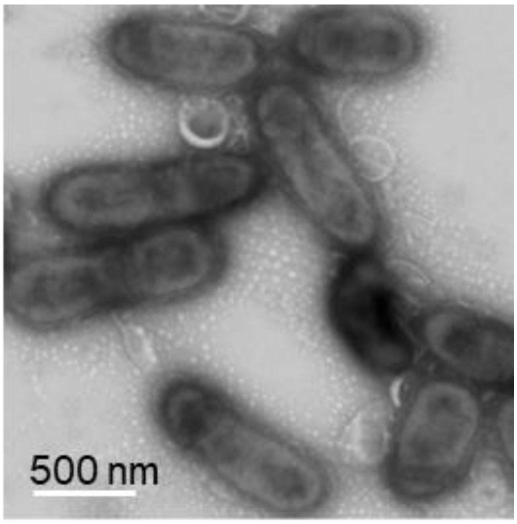

Fig. S4. Morphology of strain 20NA77.7^T. Transmission electron micrograph: bar, 500 nm. The cells were grown on R2A at 25°C for 3 days.

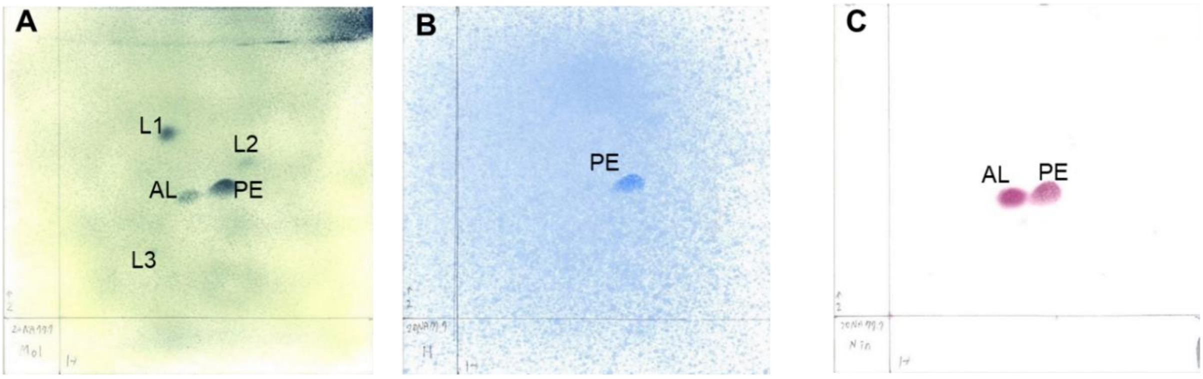

Fig. S5. Two-dimensional thin layer chromatography of polar lipids extracted from strain 20NA77.7^T. PE, phosphatidylethanolamine; AL, unidentified aminolipids; L, unidentified lipids.
